# HK2-mediated Glycolysis Inhibits Mineralization of Cementoblasts Under Compression by Suppressing the Piezo1/Wnt Signaling

**DOI:** 10.7150/ijms.109287

**Published:** 2025-07-11

**Authors:** Zhilong Huang, Hengyu Hu, Ye Meng, Houxuan Li, Lang Lei

**Affiliations:** 1 Nanjing Stomatological Hospital, Affiliated Hospital of Medical School, Institute of Stomatology, Nanjing University, Nanjing, China.; 2 Central Laboratory of Stomatology, Nanjing Stomatological Hospital, Affiliated Hospital of Medical School, Institute of Stomatology, Nanjing University, Nanjing, China.

**Keywords:** root resorption, cementoblasts, HK2-mediated glycolysis, Piezo1, Wnt/β-catenin signaling

## Abstract

**Background:** Orthodontically induced inflammatory root resorption (OIIRR) is a prevalent and severe complication during orthodontic tooth movement (OTM). Glycolysis plays a crucial role in the inflammatory responses. This study aimed to improve the cell compression model and investigate whether Hexokinase 2 (HK2)-mediated glycolysis regulates cementoblasts' mineralization through the mechanosensitive Piezo1/Wnt signaling under compressive force.

**Methods:** Mouse cementoblasts (OCCM-30) were cultured under compressive force with different buffer membranes to mimic the periodontal membrane. The flow cytometry and CCK-8 assay were utilized to evaluate cell apoptosis and viability. Piezo1 and HK2 were knocked down by small interfering RNA (siRNA). The level of Wnt/β-catenin signaling was detected by qRT-PCR and Western blotting, and the cellular localization of β-catenin was detected by immunofluorescence staining.

**Results:** The viability and apoptosis of cementoblasts showed no significant change under compression at 2.0 g/cm^2^ for 12 hours with Polytetrafluoroethylene (PTFE) buffer membrane. HK2-mediated glycolysis was increased in compressed cementoblasts with elevated ratio of the receptor activator of nuclear factor kappa-B ligand/osteoprotegerin (RANKL/OPG) and decreased expression of Piezo1 and mineralization-related markers. The Piezo1 activated Wnt signaling by increasing the nuclear translocation of β-catenin, which increased the levels of mineral-related markers. Whereas, knockdown of Piezo1 showed the opposite trend. Knockdown of HK2 to inhibit glycolysis partially reversed the compression-induced decline in Piezo1 and mineralization-related markers, as well as the rise in the RANKL/OPG ratio.

**Conclusions:** The cell compression model with PTFE buffer membrane effectively reduced cell damage. HK2-mediated glycolysis inhibited mineralization and enhanced osteoclast induction in cementoblasts under compression by suppressing the mechanosensitive Piezo1/Wnt signaling.

## Introduction

Orthodontically induced inflammatory root resorption (OIIRR) is a prevalent and severe complication in the orthodontic treatment 1, 2. The incidence of OIIRR varies from 56%-82% in the traditional edgewise appliances to 46%-56% in the clear aligners 3-5. Severe OIIRR may result in shortened root length, decreased tooth stability, and even the loss of natural teeth. Increased resorption of cementum by osteoclasts and decreased repair of root surfaces by cementoblasts are two principal mechanisms of OIIRR. Under mechanical compressive stress, mechanosensitive cementoblasts experience impaired mineralization capability with suppression of mineralization-related genes, such as osterix and osteopontin 6, 7.

Glycolysis is a metabolic process that transforms glucose into pyruvate and is regulated by several glycolytic enzymes. Although glycolysis is less efficient in the ATP production, it rapidly provides metabolic intermediates to meet the energy and nutritional demands of cells 8. Hexokinase 2 (HK2), the initial rate-limiting enzyme in the glycolytic process, is responsible for phosphorylating glucose into glucose-6-phosphate 9. HK2 is upregulated in a wide variety of inflammatory diseases. For example, in periodontitis, stimulation by *Porphyromonas gingivalis* enhances HK2-mediated glycolysis in osteoblasts, which subsequently promotes RANKL production and osteoclastogenesis 10. Unlike periodontitis, orthodontic tooth movement (OTM) is a sterile inflammation due to mechanical forces, potentially resulting in sterile inflammatory bone resorption and root resorption 11, 12. Therefore, we hypothesize that HK2-mediated glycolysis may also play a significant role in sterile inflammatory root resorption. However, there are currently no studies investigating how HK2-mediated glycolysis affects cementoblasts' mineralization under compression.

The Piezo1 channel is a mechanically sensitive and non-selective cation channel. When activated by mechanical stretch or shear stress, Piezo1 allows Ca²⁺ influx, converting mechanical signals into intracellular biochemical signals, activating specific pathways, such as NF-κB and MAPK signaling 13-18. Piezo1 can mediate mechanical force and regulate mineralization in periodontal ligament stem cells 19. The Wnt/β-catenin signaling can transduce mechanical stimuli 20-25. For instance, it is involved in alveolar bone remodeling during OTM, regulating the expression of osteogenic genes and contributing to osteoclastogenesis by modulating the RANKL/OPG ratio 26, 27. Based on these findings, we hypothesize that Piezo1 may mediate mechanotransduction in cementoblasts and potentially regulate cementogenesis through the Wnt/β-catenin signaling, although the hypothesis necessitates further validation. Furthermore, apart from mechanical stimulation, the expression level of Piezo1 may also be influenced by factors such as LPS-induced inflammation and high glucose concentration 28, 29. Therefore, considering that HK2-mediated glycolysis may also play a significant role in sterile inflammatory root resorption during OTM, we further hypothesize that HK2-mediated glycolysis may participate in the regulation of Piezo1/Wnt pathway. Nevertheless, there are currently no studies on this aspect.

In this study, we improved the cell compression model by applying different buffer membranes to partially mimic the periodontal ligament *in vivo*, which significantly reduced cell damage. Subsequently, we elucidated how compressive force affected mineralization and glycolysis, and investigated the function of HK2-mediated glycolysis in mineralization. Based on these findings, we further explored the connection between HK2-mediated glycolysis and the downstream molecules involved in cementoblasts' mineralization, including the Piezo1 and Wnt/β-catenin pathway. These discoveries expand our insights into the potential mechanisms governing cementoblasts' mineralization under compression, and they may aid in developing strategies to reduce the occurrence of OIIRR and to create therapeutic approaches for repairing OIIRR.

## Materials and Methods

### Cell culture and reagents

The immortalized murine cementoblast cell line, OCCM-30, was supplied by Dr. Martha J. Somerman. The proliferation medium (PM) comprised Dulbecco's modified Eagle medium (DMEM, Gibco, USA), 10% fetal bovine serum (FBS, Gibco, USA), and 1% penicillin-streptomycin (Invitrogen, USA) 30. To promote cementoblasts' mineralization, we formulated mineralization medium (MM) using PM with 0.1uM dexamethasone, 50 µg/mL ascorbic acid and 10mM sodium β-glycerophosphate (Sigma-Aldrich, USA) 31. Cementoblasts were cultured in PM or MM in a moist condition at 37°C with 5% CO2. Yoda1 (Sigma-Aldrich, USA) was used to specifically activate the Piezo1 channel. The Wnt/β-catenin signaling was particularly activated with LiCl and inhibited with Dickkopf-related protein 1 (DKK1, MedChemExpress, USA).

### Application of compressive force

**Figure 1:**
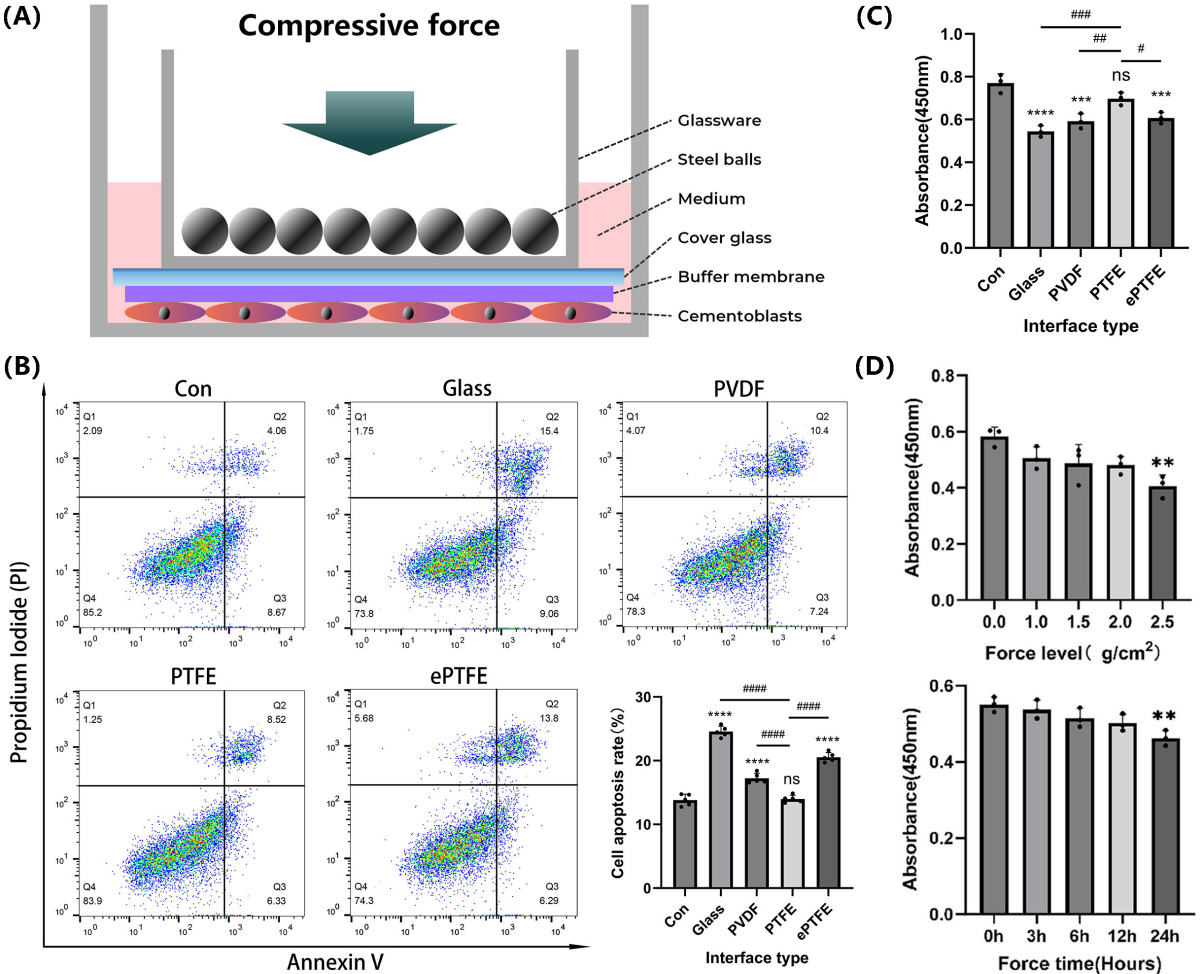
** Effects of the improved compression model on apoptosis and viability of cementoblasts. (A)** Schematic diagram of the improved compression model. **(B)** and **(C)** Cementoblasts were exposed to 2.0 g/cm² compressive force for 12 hours under different buffer membranes, and apoptosis ratio and cell viability were detected by flow cytometry with Annexin V-FITC and PI staining, and CCK-8 assay, respectively. **(D)** Cell viability of cementoblasts was evaluated by the CCK-8 assay after being exposed to different compressive forces (0, 1.0, 1.5, 2.0, and 2.5 g/cm²) for 12 hours, and under 2.0 g/cm² compression for different durations (0, 3, 6, 12, and 24 hours). The data are presented as mean ± SD ( */# P < 0.05; **/##P < 0.01; ***/###P < 0.001; ****/####P < 0.0001).

Cementoblasts were seeded into six-well or twelve-well plates. Compressive force was applied to the cells when they achieved 80-90% confluence (Figure 1a). First, we calculated the area of the cover glass placed above the cells (cm^2^) and weighed the mass of each steel bead and the mass of the glass dish holding the beads (g). Second, we adjusted the compressive force applied to the cells by controlling the number of steel beads (g/cm^2^) 31. For specific experiments, an additional 0.2 mm thick polyvinylidene fluoride (PVDF, Membrane Solutions, USA), polytetrafluoroethylene (PTFE, hydrophilicity 17.47°, Membrane Solutions, USA), or expanded polytetrafluoroethylene (ePTFE, Membrane Solutions, USA) membrane with pore diameter of 0.22um was placed under the cover glass as a buffer. OCCM-30 cells were subjected to compressive forces of 0, 1.0, 1.5, 2.0, or 2.5 g/cm² for 3, 6, 12, or 24 hours to determine the appropriate loading parameters. In experiments exploring mineralization, the cells were cultured in MM for 3 more days after compressive force application.

### Cell apoptosis assay

The ABflo® 488 Annexin V/PI apoptosis detection kit (ABclonal, China) was used to assess apoptosis. Briefly, after resuspending the cells in 500 µL of binding buffer, they were stained for 15 minutes at room temperature using 5 µL of Annexin V-FITC and 5 µL of PI. FACSCalibur flow cytometer (BD Biosciences, USA) was used to evaluate the samples, and FlowJo software (v10.8.1) was used to process the results.

### Cell viability assay

The Cell Counting Kit-8 (Beyotime Biotechnology, China) was utilized to measure the cell vitality. Briefly, after specific interventions, 10 µL of CCK-8 reagent was added to each well of 96-well plates, and the cells were incubated at 37°C with 5% CO2 for 1 hour. The absorbance at 450 nm was then measured by a multimode microplate reader for further analysis.

### Piezo1 and HK2 knockdown

Lipofectamine 3000 (Invitrogen, USA) was applied to transfect siRNA at a dose of 100 nM in Opti-MEM medium (Gibco, USA). The siRNA sequences of Piezo1 and HK2 were manufactured by an enterprise (Biosyntech, China). After co-culturing the siRNA with cells for 72 hours, we confirmed knockdown effectiveness with qPCR and WB, and the siRNA with the most significant knockdown effect was selected for subsequent experiments.

### Alkaline phosphatase (ALP) staining

The BCIP/NBT staining kit (Beyotime Biotechnology, China) was used for ALP staining. Briefly, after culturing cementoblasts in MM for 2 week, the cells were fixed in 4% paraformaldehyde for 10 minutes, followed by treatment with ALP staining buffer for 1 hour. Finally, the staining results were captured by a Nikon Ti2 microscope (Nikon, Japan). ImageJ software was used to calculate staining density for quantification.

### Von Kossa (VK) and Alizarin red S (ARS) staining

The mineralized nodules formed by cementoblasts were stained by Von Kossa staining kit (Bestbio, China) and ARS staining solution (Cell Research, China). Briefly, after culturing cementoblasts in MM for 3 weeks, the cells were fixed in 4% paraformaldehyde for 10 minutes, followed by treatment with ARS staining buffer for 20 minutes. Finally, the staining results were captured by a Nikon Ti2 microscope (Nikon, Japan). The staining density of the mineralized nodules was calculated for quantification utilizing ImageJ software.

### Glucose consumption assay and lactate production assay

Glucose and lactate concentrations were measured by the glucose (HK) assay kit (Beyotime Biotechnology, China) and the lactate assay kit (Eton Biosciences, USA), respectively. Briefly, 50 µL of cell culture supernatant was mixed with 100 µL of reaction solution at 37°C for 30 minutes, and the absorbance was measured at 340 nm and 490 nm, respectively. Glucose and lactate concentrations were calculated based on standard curves. The results were normalized to the cell count.

### Quantitative real-time polymerase chain reaction (qRT-PCR)

Total RNA was extracted from cementoblasts by the SteadyPure RNA Extraction Kit (Accurate Biotechnology, China). The RNA was reverse transcribed into cDNA by the Evo M-MLV Reverse Transcription Kit (Accurate Biotechnology, China). Following the manufacturer's protocol, the cDNA was then mixed with primers (GenScript, China) and SYBR Green/ROX qPCR Master Mix (Accurate Biotechnology, China) and subjected to qRT-PCR using the ViiA 7^TM^ Real-Time PCR System (Applied Biosystems, USA). The standard qPCR cycling conditions were 10 minutes at 95°C, followed by 40 cycles of 15 seconds at 95°C and 1 minute at 60°C. β-actin was used as the internal control, and the data were analyzed using the 2^(-ΔΔCt) method. The results were presented as the relative expression ratio of the target genes to the gene of internal control. The sequences of all primers are listed in Table 1.

### Western blotting (WB)

The cementoblasts were lysed on ice for 15 minutes using RIPA lysis buffer (Beyotime Biotechnology, China). Protein concentrations were determined with the BCA Protein Assay Kit (Beyotime Biotechnology, China). Equal amounts of protein were then separated by 4-20% sodium dodecyl sulfate-polyacrylamide gel electrophoresis (SDS-PAGE, Tiandirenhe, China) and transferred to polyvinylidene fluoride (PVDF) membranes (Millipore, USA). The PVDF membranes were blocked at room temperature with 5% bovine serum albumin (BSA) for 1 hour, followed by incubation overnight at 4°C with primary antibodies against Piezo1 (1:1000, ABclonal, China), GLUT1 (1:5000, ABclonal, China), HK2 (1:1000, Abcam, UK), PFKFB3 (1:2000, Abcam, UK), LDHA (1:2000, Abcam, UK), β-catenin (1:1000, ABclonal, China), TCF1 (1:1000, ABclonal, China), LEF1 (1:1000, ABclonal, China), RANKL (1:1000, Proteintech, USA), OPG (1:1000, Proteintech, USA), Runx2 (1:1000, Cell Signaling Technology, USA), OSX (1:1000, Cell Signaling Technology, USA), and OPN (1:1500, Cell Signaling Technology, USA), all from rabbit sources. After washing, the membranes were incubated at room temperature for 1 hour with an anti-rabbit secondary antibody (1:10000, ABclonal, China). Finally, the PVDF membranes were exposed to ECL reagent (Vazyme, China) to detect signals. Images were captured by the Tanon 5200 Chemiluminescent Imaging System (Tanon, China), and band intensities were quantified with ImageJ software after normalization to β-actin.

### Immunofluorescence (IF) staining

Cell samples were fixed with 4% paraformaldehyde, permeabilized with 0.1% Triton X-100, and blocked with 5% BSA at room temperature for 1 hour. Primary antibodies against Piezo1 (1:200, ABclonal, China), HK2 (1:200, Abcam, UK), and β-catenin (1:200, ABclonal, China) were used to specifically bind their respective target proteins, followed by visualization of the target proteins with fluorophore-conjugated secondary antibodies (1:300, ABclonal, China). The cells were stained with 4',6-diamidino-2-phenylindole (DAPI, Beyotime Biotechnology, China). Images were captured by a Nikon Ti confocal imaging system (Beyotime Biotechnology, China).

### Statistical analysis

GraphPad Prism software was used to perform Student's t-test to analyze differences between the treatment and control groups. One-way analysis of variance (ANOVA) was used for comparisons among multiple groups, followed by Tukey's post-hoc test. All experiments were independently conducted at least three times, and all data are presented as mean ± standard deviation (SD). A two-tailed P value of < 0.05 was regarded as statistically significant.

## Results

### Effects of the improved compression model on apoptosis and viability of cementoblasts

Compared to the non-compressed control group, the apoptosis rate significantly increased and cell viability dramatically diminished in the groups exposed to compression by the cover glass, PVDF, and ePTFE buffer membranes, while no significant difference was observed in the PTFE buffer membrane group (Fig. 1B-C). Following a 12-hour compression of cementoblasts with different forces (0, 1.0, 1.5, 2.0, and 2.5 g/cm²), cell viability significantly decreased in the 2.5 g/cm² group. When subjected to different durations of compression with 2.0 g/cm² (0, 3, 6, 12, and 24 hours), cell viability significantly decreased in the 24-hour group (Fig. 1D). Therefore, in this study's subsequent compression experiments, cementoblasts were exposed to a load of 2.0 g/cm² for 12 hours with a PTFE buffer membrane.

### Compression promoted glycolysis in cementoblasts

**Figure 2:**
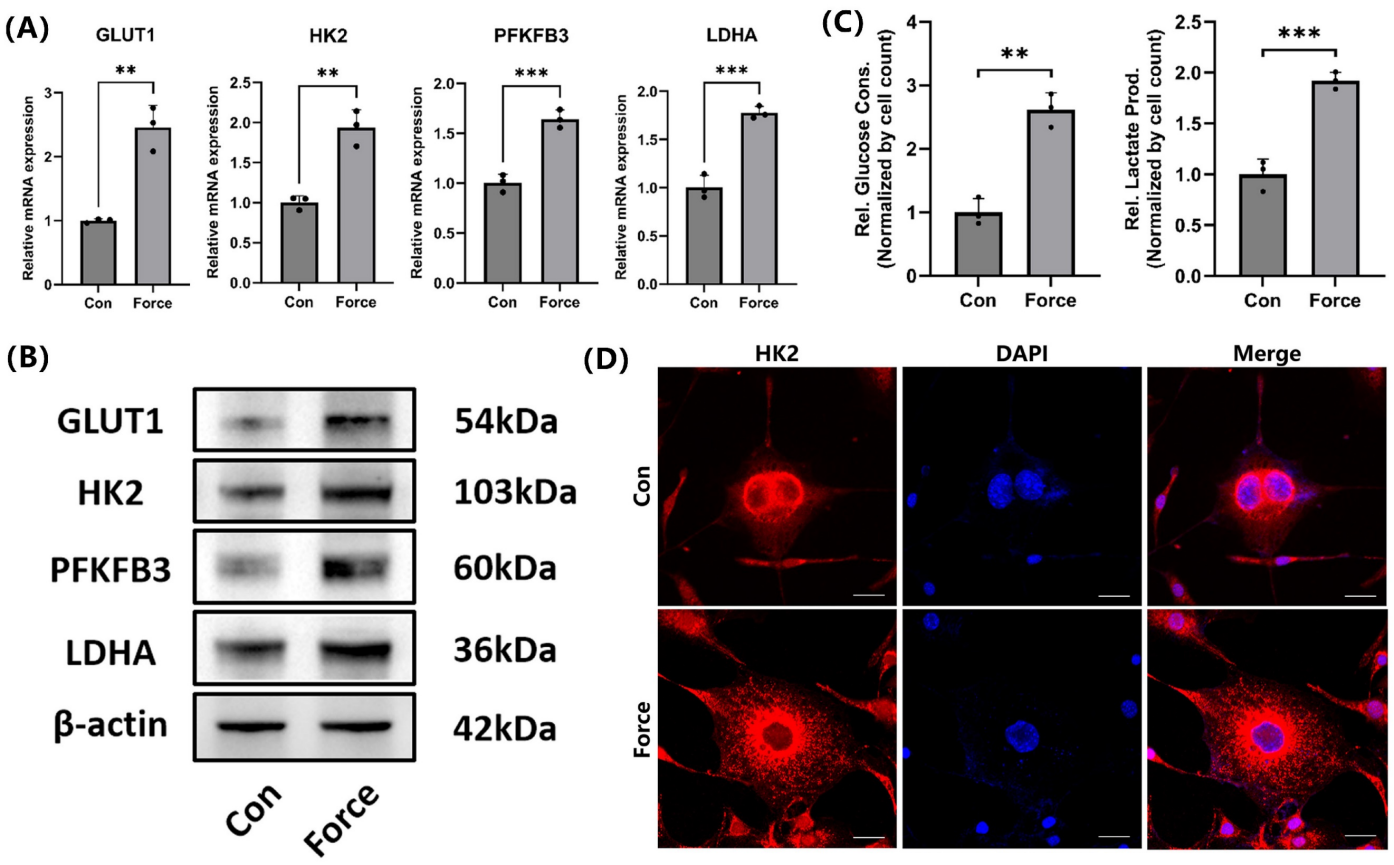
** Compression promoted glycolysis in cementoblasts. (A)** and **(B)** The relative mRNA and protein levels of GLUT1, HK2, PFKFB3, and LDHA were determined by qRT-PCR and WB. The blot images represented data from 3 independent experiments. **(C)** Glucose consumption and lactate production in cementoblasts, normalized to cell count. **(D)** Representative IF staining of HK2 with DAPI counterstaining. Scale bar = 20μm. The data are presented as mean ± SD (*P < 0.05; **P < 0.01; ***P < 0.001; ****P < 0.0001).

Under compression, the mRNA and protein levels of key glycolysis-related genes in cementoblasts, including GLUT1, HK2, PFKFB3, and LDHA, increased (Fig. 2A-B). Additionally, glucose consumption in cementoblasts increased under compression, with a corresponding rise in lactate production (Fig. 2C). IF staining demonstrated HK2 was primarily localized in the cytoplasm around the nucleus, and its expression increased under compression (Fig. 2D). These findings suggested that compression enhanced glucose demand and increased glycolysis levels in cementoblasts.

### Compression inhibited cementoblasts' mineralization and Piezo1 expression

**Figure 3:**
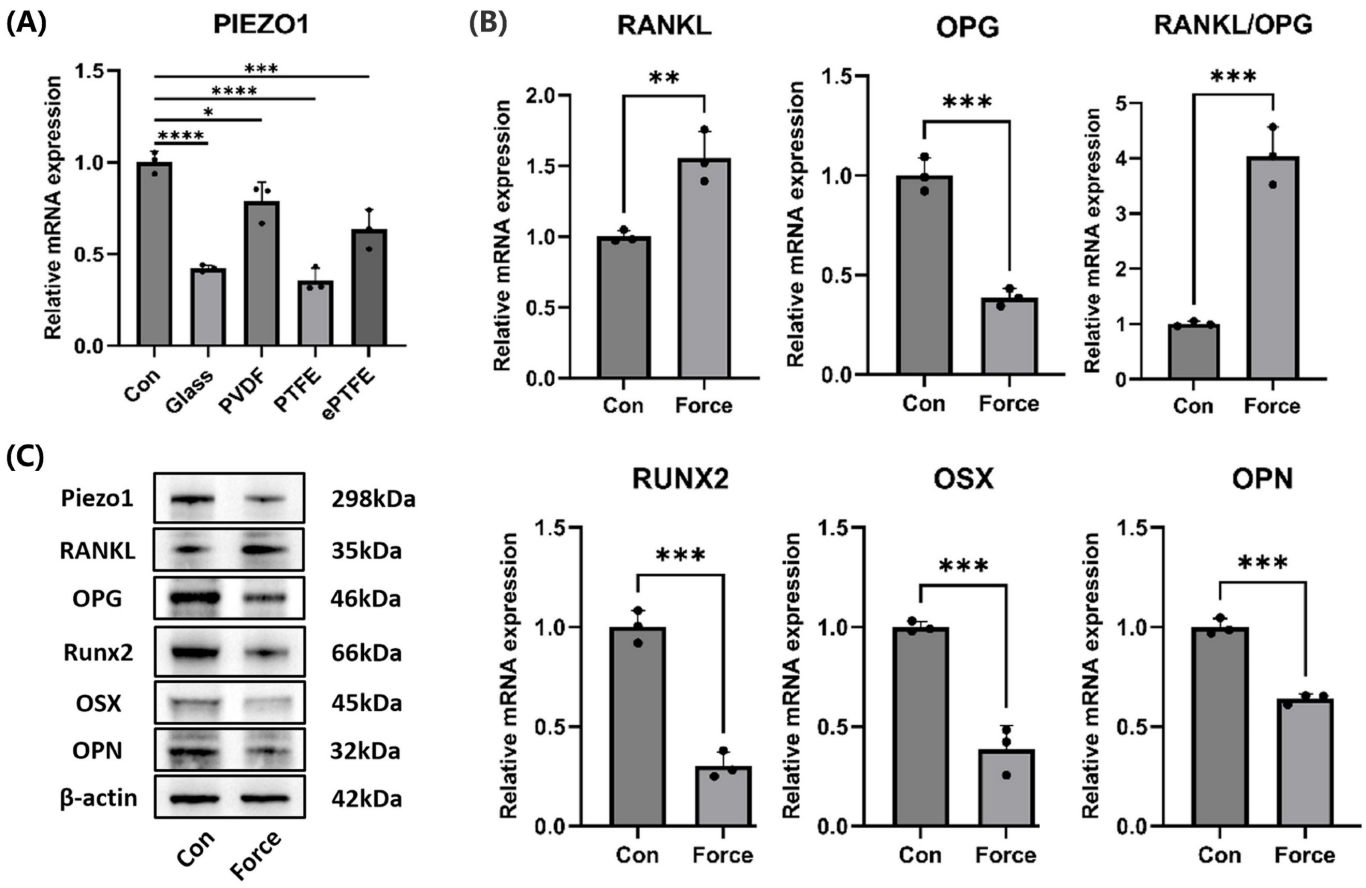
** Compression inhibited cementoblasts' mineralization and Piezo1 expression. (A)** The relative mRNA levels of Piezo1 were detected by qRT-PCR after cementoblasts were subjected to 2.0 g/cm² compression for 12 hours under distinct buffer membranes. **(B)** and **(C)** The relative mRNA and protein levels of Piezo1, RANKL, OPG, RANKL/OPG, Runx2, OSX, and OPN were identified by qRT-PCR and WB. The blot images represented data from 3 independent experiments. The data are presented as mean ± SD (*P < 0.05; **P < 0.01; ***P < 0.001; ****P < 0.0001).

Under compression, the mRNA levels of the Piezo1 in cementoblasts significantly decreased, whether compressed with the cover glass, PVDF, PTFE, or ePTFE as a buffer membrane (Fig. 3A). Western blot also showed a reduction in Piezo1 protein levels under compression (Fig. 3C). Additionally, compression led to an increase in the osteoclast-inducing molecule RANKL and a decrease in OPG, resulting in an upregulation of the RANKL/OPG ratio (Fig. 3B-C). Moreover, the mRNA and protein levels of mineralization-related molecules Runx2, OSX, and OPN decreased (Fig. 3B-C). These results suggested that compression inhibited cementoblasts' mineralization and Piezo1 expression, while enhancing osteoclast induction.

### Piezo1 mediated mechanotransduction in cementoblasts and promoted mineralization

**Figure 4:**
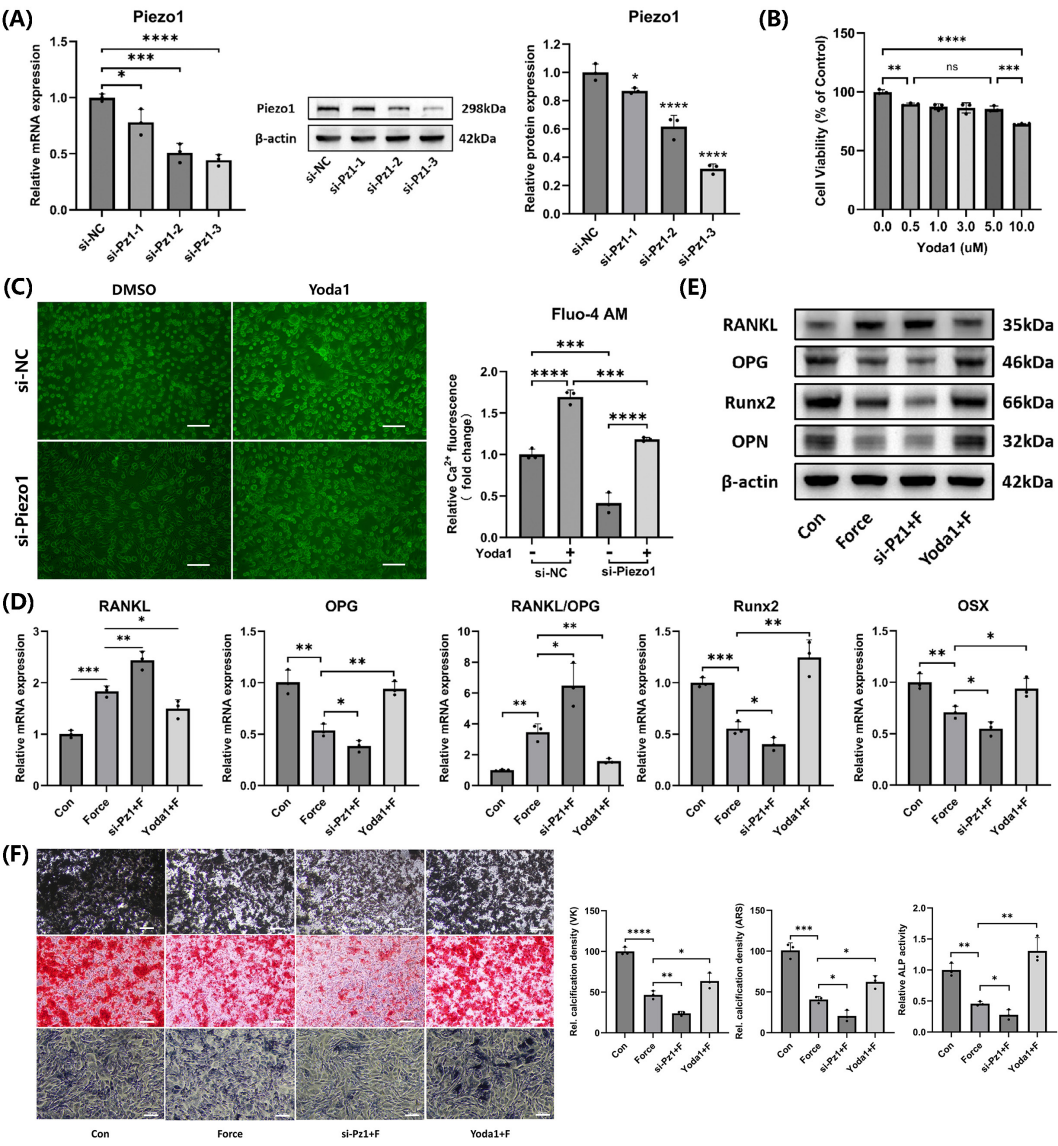
** Piezo1 mediated mechanotransduction in cementoblasts and promoted mineralization. (A)** The knockdown efficiency of the three Piezo1 siRNAs was confirmed using qRT-PCR and WB. **(B)** The impact of varying doses of Yoda1 on cell viability after 24h was evaluated by the CCK-8 assay. **(C)** Intracellular calcium concentration was detected by the Fluo-4 AM calcium ion fluorescence probe. Images were captured by fluorescence microscopy. Scale bar = 50μm. **(D)** and **(E)** The relative mRNA and protein levels of RANKL, OPG, RANKL/OPG, Runx2, OSX, and OPN were identified using qRT-PCR and WB. The blot images represented data from three independent experiments. **(F)** Von Kossa (VK), Alizarin Red S (ARS) (after 3 weeks of mineralization induction) and alkaline phosphatase staining (after 2 weeks of mineralization induction). Scale bar = 10μm. The data are presented as mean ± SD (*P < 0.05; **P < 0.01; ***P < 0.001; ****P < 0.0001). Con: Control, Pz1: Piezo1, F: Force.

Three different Piezo1 siRNA sequences were used to knock down Piezo1 expression. Both mRNA and protein levels showed that the si-Pz1-3 had the most significant knockdown effect (Fig. 4A). Therefore, si-Pz-3 was selected for subsequent experiments. The CCK-8 assay indicated that Yoda1, at doses between 0.5 µM and 5.0 µM, exhibited no significant impact on cell viability after 24 hours (Fig. 4B). As a result, 5.0 µM Yoda1 for 24 hours was chosen for further experiments. Intracellular calcium levels were measured using the Fluo-4 AM calcium ion fluorescence probe, which confirmed that si-Piezo1 decreased and Yoda1 increased intracellular calcium levels, verifying the effectiveness of si-Piezo1 and Yoda1 (Fig. 4C). Compared to the group subjected to compression alone, the group with Piezo1 knockdown via siRNA followed by compression showed an elevation in the RANKL/OPG ratio and a reduction in Runx2, OSX, and OPN expression. In contrast, activating Piezo1 with Yoda1 before compression produced opposite results (Fig. 4D-E). Furthermore, Von Kossa (VK), Alizarin Red S (ARS) and ALP staining in cementoblasts were consistent with the findings above (Fig. 4F). Therefore, the findings revealed Piezo1 mediated mechanotransduction in cementoblasts, promoting mineralization while inhibiting osteoclast induction.

### Piezo1 activated the Wnt/β-catenin signaling

**Figure 5:**
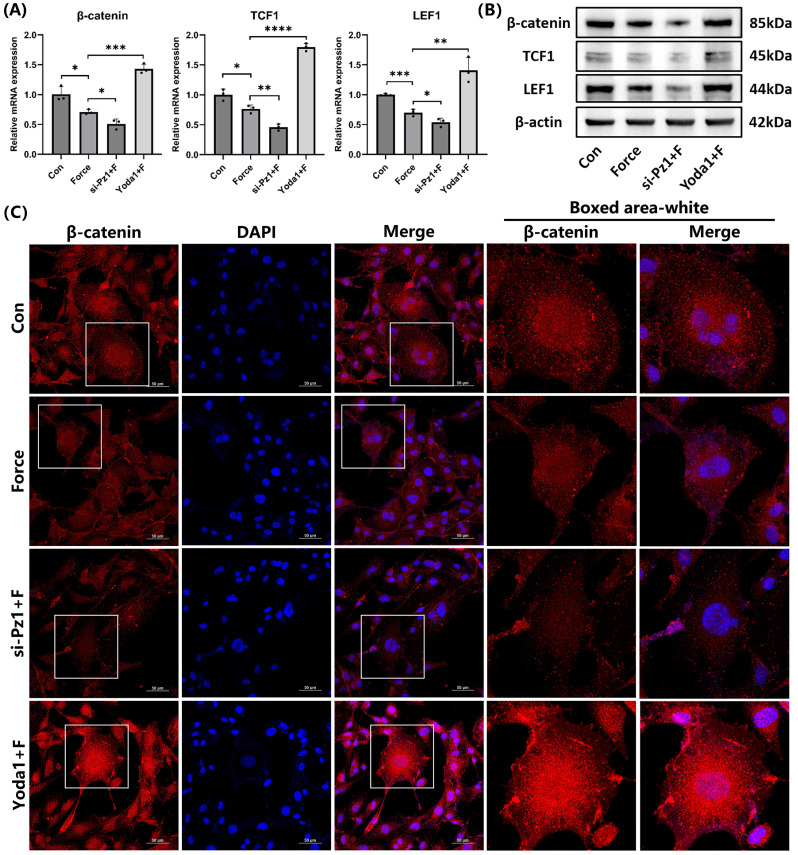
**Piezo1 activated the Wnt/β-catenin signaling. (A)** and **(B)** The relative mRNA and protein levels of β-catenin and its target transcription factors TCF1 and LEF1 were identified by qRT-PCR and WB. The blot images represented data from 3 independent experiments. **(C)** Immunofluorescence of β-catenin and its localization with DAPI counterstaining. Scale bar = 50μm. The data are presented as mean ± SD (*P < 0.05; **P < 0.01; ***P < 0.001; ****P < 0.0001). Con: Control, Pz1: Piezo1, F: Force.

The relative mRNA and protein levels of the key signaling molecule β-catenin and its target transcription factors TCF1 and LEF1 in the canonical Wnt/β-catenin pathway decreased under compression. This trend became more pronounced when Piezo1 was knocked down with siRNA. However, when Piezo1 was activated by Yoda1, the trend was reversed (Fig. 5A-B). IF staining of β-catenin was consistent with these results, and its nuclear translocation significantly increased when Piezo1 was activated (Fig. 5C). These findings indicated that Piezo1 positively regulated the Wnt/β-catenin signaling.

### Wnt/β-catenin signaling promoted cementoblasts' mineralization

**Figure 6:**
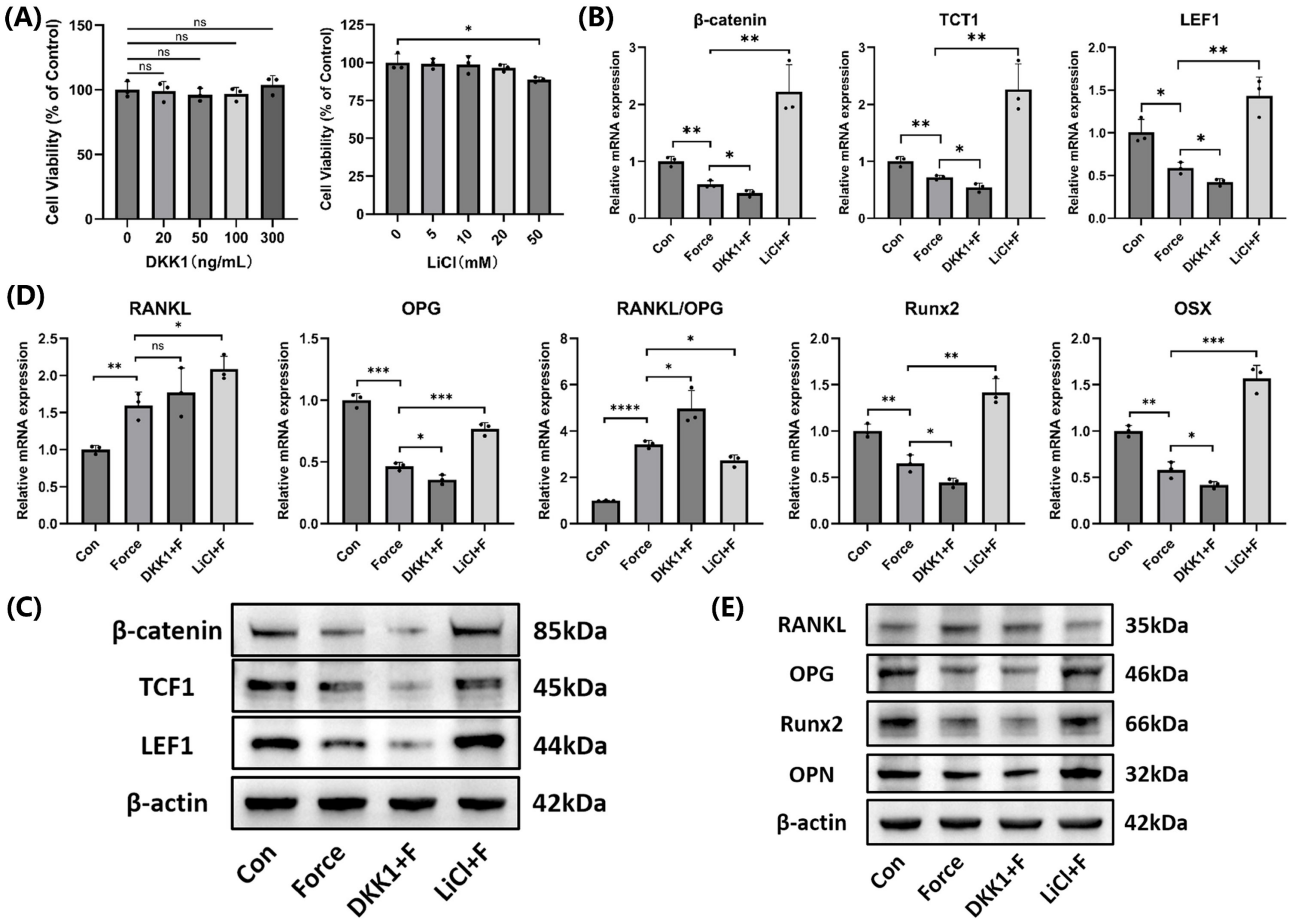
** The Wnt/β-catenin signaling promoted mineralization of cementoblasts. (A)** The effects of different concentrations of DKK1 and LiCl on cell viability after 24 hours was evaluated using the CCK-8 assay. **(B)** and **(C)** The relative mRNA and protein levels of β-catenin and its target transcription factors TCF1 and LEF1 were detected by qRT-PCR and WB. **(D)** and **(E)** The relative mRNA and protein levels of RANKL, OPG, RANKL/OPG, Runx2, OSX, and OPN were identified by qRT-PCR and WB. The blot images represented data from 3 independent experiments. The data are presented as mean ± SD (*P < 0.05; **P < 0.01; ***P < 0.001; ****P < 0.0001). Con: Control, F: Force.

After treatment with 300 ng/mL DKK1 and 20 mM LiCl for 24 hours, no significant effects on cell viability were observed, and these parameters were used in subsequent experiments (Fig. 6A). The effectiveness of the Wnt/β-catenin pathway inhibitor DKK1 and activator LiCl was confirmed at both mRNA and protein levels (Fig. 6B-C). Compared to the group subjected to compression alone, the DKK1-treated group showed an elevation in the RANKL/OPG ratio and a reduction in Runx2, OSX, and OPN expression, while the LiCl-treated group exhibited the opposite results (Fig. 6D-E). The findings indicated the Wnt/β-catenin signaling positively regulated mineralization but inhibited osteoclast induction in cementoblasts.

### HK2-mediated glycolysis inhibited cementoblasts' mineralization by suppressing Piezo1 expression

**Figure 7:**
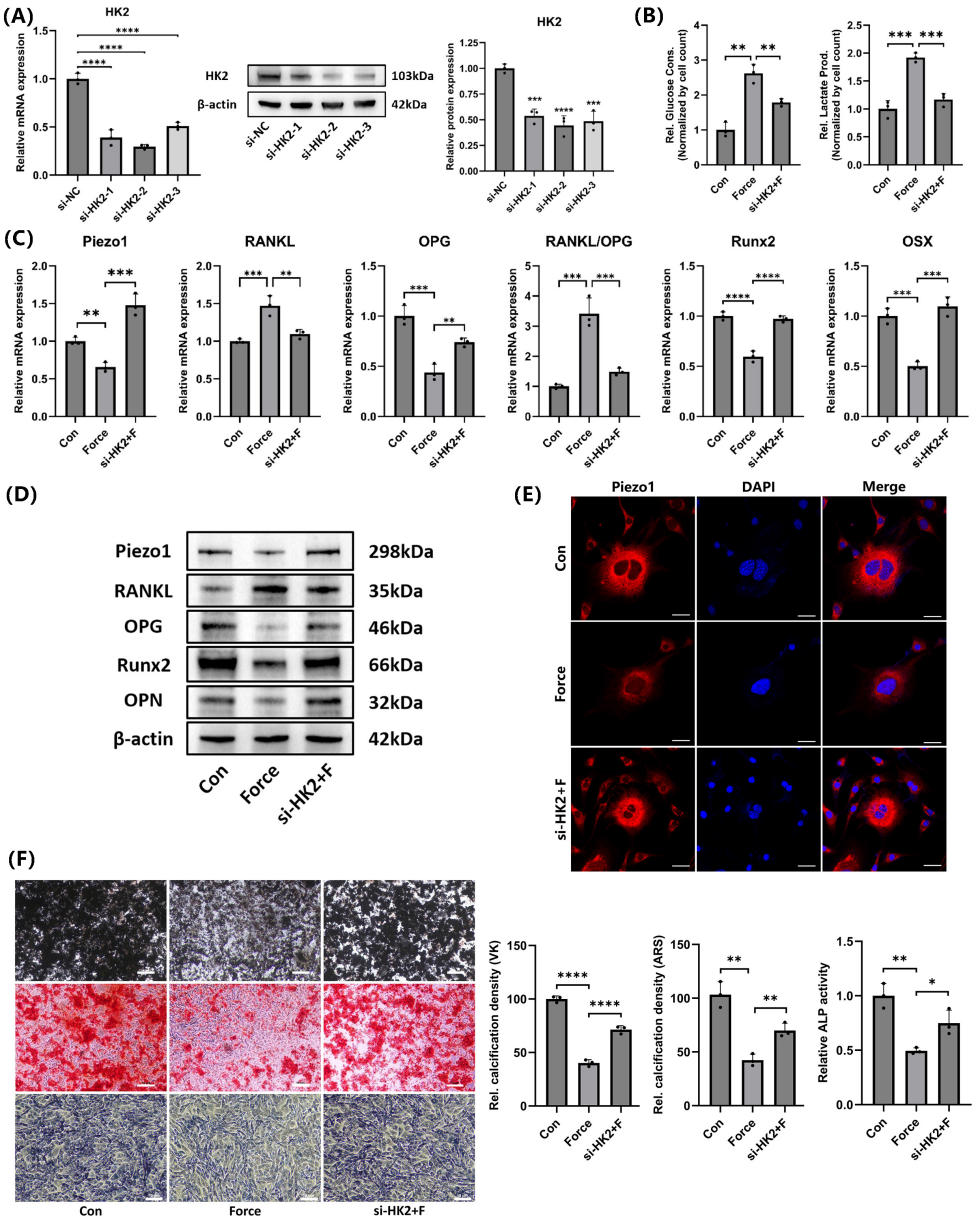
**HK2-mediated glycolysis inhibited cementoblasts' mineralization by suppressing Piezo1 expression. (A)** The knockdown efficiency of the three HK2 siRNAs was verified by qRT-PCR and WB. **(B)** Glucose consumption and lactate production in cementoblasts, normalized to cell number. **(C)** and **(D)** The relative mRNA and protein levels of Piezo1, RANKL, OPG, RANKL/OPG, Runx2, OSX, and OPN were identified by qRT-PCR and WB. The blot images represented data from 3 independent experiments. **(E)** Immunofluorescence of Piezo1 with DAPI counterstaining. Scale bar = 20μm. **(F)** Von Kossa (VK), Alizarin Red S (ARS) (after 3 weeks of mineralization induction) and alkaline phosphatase staining (after 2 weeks of mineralization induction). Scale bar = 10μm. The data are presented as mean ± SD (*P < 0.05; **P < 0.01; ***P < 0.001; ****P < 0.0001). Con: Control, F: Force.

Three different HK2 siRNA sequences were used to knock down HK2 expression. Both mRNA and protein levels showed the si-HK2-2 had the most significant knockdown effect (Fig. 7A). Therefore, si-HK2-2 was selected for subsequent experiments. Glucose consumption and lactate production measurements further confirmed that si-HK2 effectively inhibited glycolysis under compression (Fig. 7B). Interestingly, si-HK2 reversed the compression-induced decrease in Piezo1 expression (Fig. 7C-D), which corresponded with the IF staining outcomes of Piezo1 (Fig. 7E). As Piezo1 expression was upregulated, the corresponding RANKL/OPG ratio and the expression levels of Runx2, OSX, and OPN were partially reversed compared to the compression group (Fig. 7C-D). Von Kossa (VK), Alizarin Red S (ARS) and ALP staining results further supported these findings (Fig. 7F). These results suggested that HK2-mediated glycolysis inhibited mineralization and enhanced osteoclast induction in cementoblasts by negatively regulating Piezo1 expression.

## Discussion

Several studies have employed devices to apply compressive force of 1.5 g/cm² for 12 hours to cells, with the common approach of placing a cover glass directly over the cells 31-33. However, since both the cover glass and the cell culture dish are rigid, this can lead to uneven force distribution and even cell death. Therefore, in this study, we improved the cell compression model by adding an extra PTFE buffer membrane above the cells to partially simulate the periodontal ligament *in vivo*. Furthermore, we validated that, compared to the traditional device using only a cover glass, the improved device significantly reduced the apoptosis rate and increased cell viability, demonstrating the superiority of our improved design. In addition, considering we used a PTFE membrane for buffering, we regard applying a compressive force of 2.0 g/cm² in our experiment is reasonable.

The decreased level of Piezo1 in cementoblasts under compression indicated a reduced ability of the cells to transmit mechanical signals, which might further affect their mineralization. It has been reported that the RANKL/OPG ratio regulates the dynamic resorption and deposition of alveolar bone, and a high RANKL/OPG ratio suggests active alveolar bone resorption, a process similar to external root resorption 34. Furthermore, Runx2, OSX, and OPN are essential regulators of cementoblasts' mineralization 35, 36. Therefore, the observed increase in the RANKL/OPG ratio and the decrease in Runx2, OSX, and OPN expression in this study suggested that the osteoclast-inducing potential of cementoblasts was enhanced under compression, while their mineralization capacity was reduced. This was consistent with the high incidence of OIIRR observed clinically.

Our research established a connection between Piezo1 and Wnt/β-catenin signaling in cementoblasts under compression. Our results indicated that Piezo1 positively regulated cementoblasts' mineralization and negatively regulated their osteoclast induction through modulating the Wnt/β-catenin signaling. However, there is controversy, as another study has found that in macrophages, Piezo1 promoted osteoclastogenesis by stimulating the production of various inflammatory factors 37. This seemingly contradictory phenomenon might be related to the functional differences between cell types and the differences in the activation of downstream signaling pathways. Cementoblasts are primarily responsible for the synthesis and mineralization of cement matrix; therefore, activation of Piezo1 may induce signaling pathways related to bone formation, such as the Wnt/β-catenin pathway. In contrast, macrophages majorly participate in immune response and inflammation regulation; therefore, Piezo1 activation may upregulate osteoclast activity through inflammation-related pathways, such as NF-κB. However, this hypothesis requires further research for validation.

Previous studies found that glycolysis was enhanced in osteoblasts during periodontitis, which promoted the production of RANKL and the generation of osteoclasts 10. Additionally, other studies showed that glycolysis is essential for osteoclast differentiation and regulates pyroptosis in cementoblasts during periodontitis 38, 39. HK2 is the initial rate-limiting enzyme in the glycolytic process and is upregulated in many diseases associated with enhanced glycolysis 9. The findings from our study on cementoblasts are consistent with these previous studies. We demonstrated that glycolysis levels in cementoblasts increased under compression, and inhibiting glycolysis with si-HK2 not only upregulated Piezo1, Runx2, OSX, and OPN expression but also decreased the RANKL/OPG ratio. This indicated that HK2-mediated glycolysis played a crucial role in OIIRR by suppressing the Piezo1/Wnt signaling. Although previous studies found that Piezo1 promoted glycolysis in macrophages 37, our study is the first to show that glycolysis, which rapidly provides metabolic intermediates to meet the energy and nutrient demands of cells, can in turn regulate Piezo1. This provides new insights into the relationship between Piezo1 and HK2-mediated glycolysis. Therefore, inhibiting HK2-mediated glycolysis or activating the Piezo1/Wnt signaling pathway may be potential strategies to reduce the occurrence of OIIRR and repair OIIRR.

Although HK2 and Piezo1 have shown potential as therapeutic targets for the prevention and treatment of OIIRR in this study, the specificity and safety of the present drug delivery systems hinder their clinical translation. HK2 and Piezo1 are widely distributed in various tissues in the body, and participate in multiple physiological processes 18, 40. Therefore, inhibitors of HK2 and Piezo1 may show off-target effects and side effect risks 41. In future studies, combining different drug delivery systems may help achieve the clinical translation of HK2 and Piezo1 specifically targeting cementoblasts.

Glucose provides ATP through pathways such as glycolysis and oxidative phosphorylation during metabolism. However, when glucose supply is insufficient, cells may turn to other energy substrates, such as glutamine and fatty acids to maintain their physiological functions 42, 43. In OIIRR, cells within the periodontal membrane may be in a nutrition deprivation-like situation due to vascular occlusion with a shortage of glucose, glutamine and fatty acids. However, presently no research has addressed this issue, and further investigation into the roles of energy substrates such as glutamine and fatty acids in OIIRR is necessary in future studies.

However, since there may be species differences between murine and human cementoblasts in terms of bone and energy metabolism, it is necessary to conduct further studies using human cementoblasts. Although we employed the improved cell compression model, it still cannot fully replicate the biological conditions of cementoblasts during OIIRR *in vivo*. Therefore, further *in vivo* studies in animal models are needed to provide more reliable evidence. Moreover, while we discovered that HK2-mediated glycolysis inhibited the Piezo1/Wnt signaling, the specific mechanism by which glycolysis exerts this inhibition requires further investigation.

## Conclusion

This study demonstrated that the cell compression model improved with PTFE buffer membrane effectively reduced cell damage. Piezo1 mediated mechanotransduction in cementoblasts. HK2-mediated glycolysis inhibited mineralization and enhanced osteoclast induction in cementoblasts under compressive force by suppressing the Piezo1/Wnt signaling. These discoveries expand our insights into the potential mechanisms governing cementoblasts' mineralization under compression and exploring the therapeutic approaches of HK2 inhibitors (e.g., 2-DG) or Piezo1 agonists (e.g., Yoda1) may assist in reducing the occurrence of OIIRR and promoting the repair of OIIRR.

## Figures and Tables

**Table 1:** Primer sequences for qRT-PCR.

Gene	Forward primer (5'-3')	Reverse primer (5'-3')
Piezo1	GCTTGCTAGAACTTCACG	GTACTCATGCGGGTTG
GLUT1	AGGGCCTAAGGTCACATGAA	AACTCCTCAATAACCTTCTGGG
HK2	TGATCGCCTGCTTATTCACGG	AACCGCCTAGAAATTCTCCAGA
PFKFB3	CTACCTCAACTGGATAGGTGTTC	AGGGCGGAAGAAGTTGTAAG
LDHA	TGTCTCCAGCAAAGACTACTGT	GACTGTACTTGACAATGTTGGGA
β-catenin	CGCCGCTTATAAATCGCTCC	TTCACAGGACACGAGCTGAC
TCF1	AGCTTTCTCCACTCTACGAACA	AATCCAGAGAGATCGGGGGTC
LEF1	CGGGAAGAGCAGGCCAAATA	AGCTTCTCTTACCACCTGAAGTC
RANKL	CAGCATCGCTCTGTTCCTGTA	CTGCGTTTTCATGGAGTCTCA
OPG	ACCCAGAAACTGGTCATCAGC	CTGCAATACACACACTCATCACT
Runx2	ATGCTTCATTCGCCTCACAAA	GCACTCACTGACTCGGTTGG
OSX	GGATCTGAGTGGGAACAAGAG	ATAGTGAGCTTCTTCCTGGGTA
OPN	CGTGAGTCCCATTAAGATGGAGT	CCCGACAGTGGATATAGAACAGA
β-actin	TAAAACCCGGCGGCGCA	ATCCATGGCGAACTGGTGG

GLUT1**:** Glucose transporter 1; PFKFB3**:** 6-phosphofructo-2-kinase/fructose-2,6-bisphosphatase 3; LDHA**:** Lactate dehydrogenase A; TCF1** -** T-cell factor 1; LEF1**:** Lymphoid enhancer-binding factor 1; Runx2**:** Runt-related transcription factor 2; OSX**:** Osterix; OPN**:** Osteopontin.
